# Microfluidic sorting of intrinsically magnetic cells under visual control

**DOI:** 10.1038/s41598-017-06946-x

**Published:** 2017-07-31

**Authors:** Ahne Myklatun, Michele Cappetta, Michael Winklhofer, Vasilis Ntziachristos, Gil G. Westmeyer

**Affiliations:** 1 Institute of Biological and Medical Imaging, Helmholtz Zentrum München, Ingolstädter Landstrasse 1, D-85764 Neuherberg, Germany; 2 Institute of Developmental Genetics, Helmholtz Zentrum München, Ingolstädter Landstrasse 1, D-85764 Neuherberg, Germany; 3 Department of Nuclear Medicine, Technical University Munich, Ismaninger Strasse 22, D-81675 Munich, Germany; 4 Institute for Biology and Environmental Sciences, University of Oldenburg, Carl-von-Ossietzky-Strasse 9–11, D-26129 Oldenburg, Germany; 5 Chair of Biological Imaging, TUM School of Medicine, Technical University of Munich, Trogerstrasse 9, D-81675 Munich, Germany

## Abstract

Magnetic cell sorting provides a valuable complementary mechanism to fluorescent techniques, especially if its parameters can be fine-tuned. In addition, there has recently been growing interest in studying naturally occurring magnetic cells and genetic engineering of cells to render them magnetic in order to control molecular processes via magnetic fields. For such approaches, contamination-free magnetic separation is an essential capability. We here present a robust and tunable microfluidic sorting system in which magnetic gradients of up to 1700 T/m can be applied to cells flowing through a sorting channel by reversible magnetization of ferrofluids. Visual control of the sorting process allowed us to optimize sorting efficiencies for a large range of sizes and magnetic moments of cells. Using automated quantification based on imaging of fluorescent markers, we showed that macrophages containing phagocytosed magnetic nanoparticles, with cellular magnetic dipole moments on the order of 10 fAm^2^, could be sorted with an efficiency of 90 ± 1%. Furthermore, we successfully sorted intrinsically magnetic magnetotactic bacteria with magnetic moments of 0.1 fAm^2^. In distinction to column-based magnetic sorting devices, microfluidic systems can prevent sample contact with superparamagnetic material. This ensures contamination-free separation of naturally occurring or bioengineered magnetic cells and is essential for downstream characterization of their properties.

## Introduction

Targetable magnetic nanostructures have been valuable tools for studying biomechanical properties of cellular structures by exerting localized forces via magnetic field gradients^1, 2^. In recent years, there has been an increasing interest in adding a level of precision by genetically altering cells such that they would become specifically responsive to magnetic fields^3–8^. There is furthermore a long-standing hypothesis that certain animals may be able to sense Earth’s magnetic field via magnetomechanical signal transduction mediated by magnetic biomineralizations attached to mechanosensitive cellular structures^9–12^, analogous to what is observed in magnetotactic bacteria^13, 14^. It is thus of interest to sort cells based on intrinsic magnetic properties in order to subject them to biophysical and chemical analyses for *e*.*g*. optimization of bioengineering applications.

For these downstream analyses it is crucial to avoid contamination with metallic materials during the enrichment step. It is furthermore desirable to have visual control of the sorting process and tunable separation parameters such that cells with a large range of sizes and magnetic moments can be sorted. Commercial magnetic sorting systems, in which a mesh of superparamagnetic particles creates strong magnetic field gradients in the presence of a permanent magnet^15^ have become a popular tool in biomedical research. However, this design introduces a risk for contamination with superparamagnetic material with which the samples come in close contact. In the case of cells dissociated from primary tissue, the large and not tunable magnetic fields can also cause magnetization and subsequent enrichment of undesired iron-rich cells, such as macrophages^16^. Furthermore, the tight spaces in the mesh, necessary to ensure short distances to the magnetized superparamagnetic material, can cause a considerable amount of unspecific mechanical entrapment of cells.

**Figure 1:**
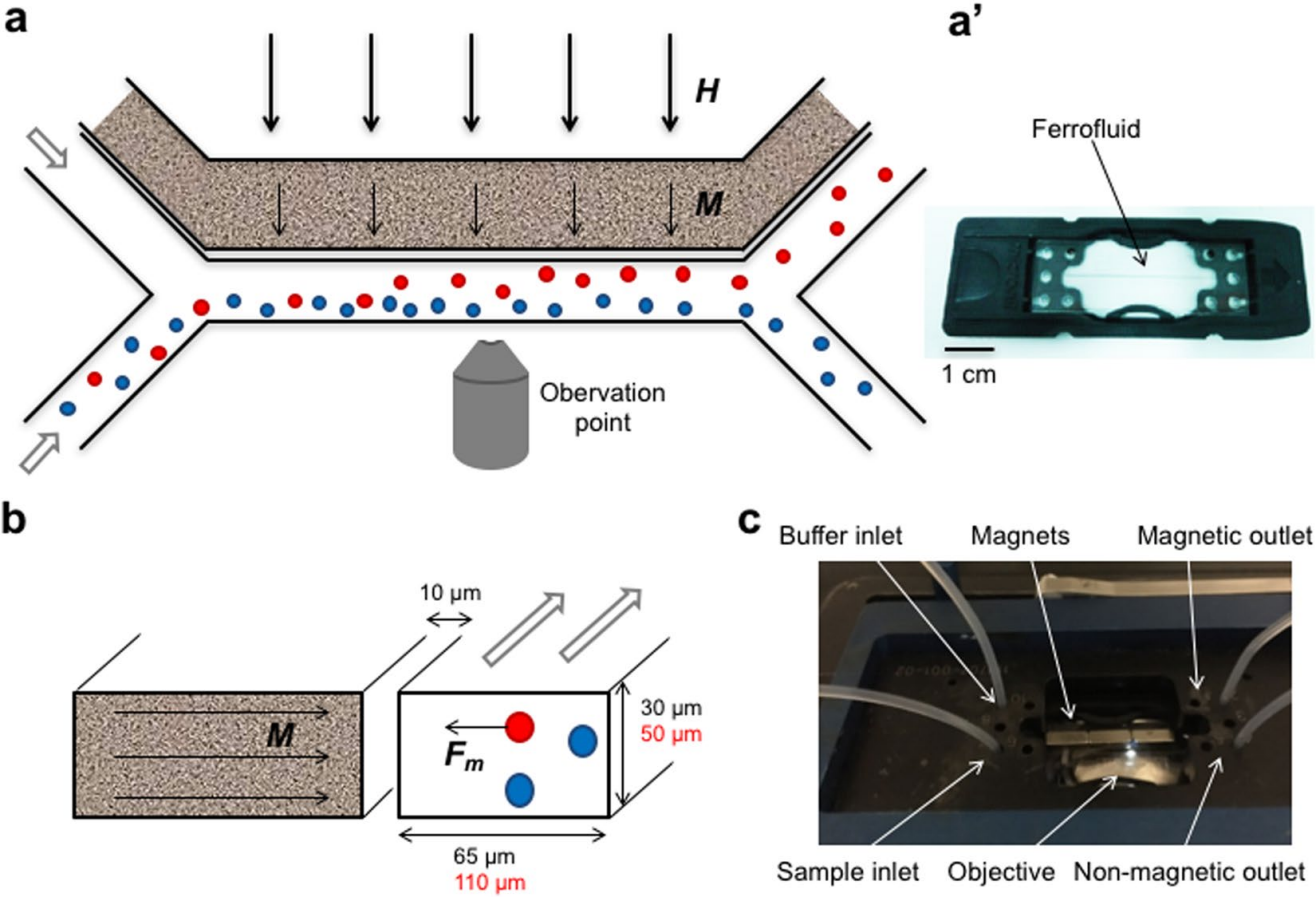
Design of microfluidic sorting device. The microfluidic device is shown from above (**a**,**a**’) and as a cross section trough the channels (**b**). A ferrofluid is filled in the side channel distanced 10 µm from the sorting channel (**a’**). In the presence of an external magnetic field (thick arrows, **H**) the nanoparticles in the ferrofluid align to magnetize the ferrofluid (thin arrows, **M**) and create a gradient field. This results in an attractive force (**F**_**m**_) acting on magnetic cells (red), whereas non-magnetic cells (blue) continue in the sample flow (**a** and **b**). The small magnetotactic bacteria were tested in a sorting channel with reduced dimensions (black) compared to dimensions used for macrophages (red). The microfluidic chip (**a’**) is placed inside a chip holder to ensure stable connection of the tubing and placed under a fluorescent microscope for online monitoring of the separation process (**c**).

The advantages of microfluidic magnetic cell separation include precise control of fluids due to laminar flow, tunable parameters for separation and visual control of the sorting process^17, 18^. In addition, these systems can be designed such that they prevent contact of the sample with magnetic materials in the sorting device. The general separation principle is based on transversal deflection of target cells from a longitudinal fluid flow. This is achieved through application of a magnetic field gradient transversal to the flow direction of the cells, which exerts a force on magnetized cells in the sorting channel (Fig. 1). The force on a given cell is proportional to its magnetic moment as well as the field gradient, which is therefore one of the key parameters in magnetic separation applications.

Precise control of the magnetic field gradient can be achieved by introducing electromagnets in the system^19, 20^. In this case however, the gradient is limited by the low amount of currents that can be applied due to the small dimensions and the associated generation of heat in the system. More often, the strong gradient necessary for efficient sorting is produced by enhancing the magnetic field from a permanent magnet through interaction with ferromagnetic material^21–26^. For instance, it has previously been shown that the presence of nickel microparticles (immersed in silicon oil) in a side channel can enhance the magnetic field gradient. The resulting magnetic force on the cells was 3.3 times greater than with a magnet alone^23^. We modified this principle, using a suspension of superparamagnetic nanoparticles (ferrofluid), which can be reversibly magnetized and retain phase stability^27^.

The reversible magnetization (*M*) of a suspension of superparamagnetic particles (typically 10 nm in the case of magnetite) in the presence of a magnetic field (*H*) is given by the Langevin function,

1 $${M}_{L}(H)=n\mu (\coth (\frac{{\mu }_{0}H\mu }{{k}_{B}T})-\frac{1}{\frac{{\mu }_{0}H\mu }{{k}_{B}T}}),$$

where *H* is the magnetizing field, *n* is the number of nanoparticles per unit volume in the ferrofluid, *µ* is the magnetic moment of each nanoparticle, *µ*_*0*_ is the permeability of free space, *k*_*B*_ is the Boltzmann’s constant and *T* is the absolute temperature. For dense ferrofluids (>10% Vol magnetic material) the particles tend to align and form chains in the presence of the magnetic field, resulting in a higher initial susceptibility. This is due to a significant contribution to the magnetization from interparticle correlations, which can be taken into consideration by introducing into equation 1 an effective magnetic field (*H*_*e*_) adding the contribution from the surrounding particles^28^. This effective magnetic field is given by

2 $${H}_{e}=H+\frac{4\pi }{3}{M}_{L}+\frac{{(4\pi )}^{2}}{144}{M}_{L}\frac{{\rm{\Delta }}{M}_{L}}{{\rm{\Delta }}H},$$

where *M*_*L*_ is the Langevin magnetization and *H* is the applied magnetizing field (Supplementary Fig. S1). The resulting magnetic field decays rapidly with distance, yielding a strong gradient of the magnetic field intensity.

In this study we optimized a microfluidic magnetic cell separation system and evaluated it for intrinsically magnetic cells. We generated the magnetic gradient by introducing a ferrofluid into a side channel in close proximity to the sample and performed simulations of the magnetic field distribution in order to characterize the expected magnetic gradients in the system. We also developed an automated video analysis for convenient quantification of the sorting efficiency. The performance was first validated with a macrophage cell line after phagocytosis of nanoparticles, yielding high cellular magnetic moments. We observed a 10-fold increase in the number of sorted cells compared to separation with the magnet alone. We also sorted magnetotactic bacteria^13, 29^, which produce biogenic magnetite crystals and thus have low intrinsic magnetic moments, to showcase that the particle-based microfluidic system could be used for efficient separation of naturally occurring magnetic cells.

## Results and Discussion

### Design of the microfluidic device and magnetic simulations

We used a design with two inlets and two outlets in which the sample containing the cells of interest was injected from one inlet and a buffer solution was added from the second inlet (Fig. 1a). By adding a magnetic field with the intensity gradient orthogonal to the direction of the flow, a magnetic force was applied to the cell, proportional to its magnetic moment and the gradient of the magnetic field intensity. This force attracted the magnetized cells across the channel towards the buffer flow, allowing for the separation of the cells of interest from the rest of the sample (Fig. 1b). In order to generate a strong gradient of the magnetic field intensity and thus increase the efficiency of the sorting process, we used a side channel in close proximity (10 µm) to the sorting channel and filled it with a ferrofluid. The distance between the sample and the magnetic particles was minimized and the width of the sorting channel was limited only by the size of the cells and the production methods (in our case the minimum width had to be twice the channel height). A reduced distance between sample and particles was shown to have a greater effect on the sorting performance compared to increasing the size of the channels, resulting in a longer interaction time of the cells with the magnetic field^26^. The interaction time is also influenced by the shape of the side channel, which we maximized by using a straight channel running in close proximity to the sorting channel along its whole length (Fig. 1a).

**Figure 2:**
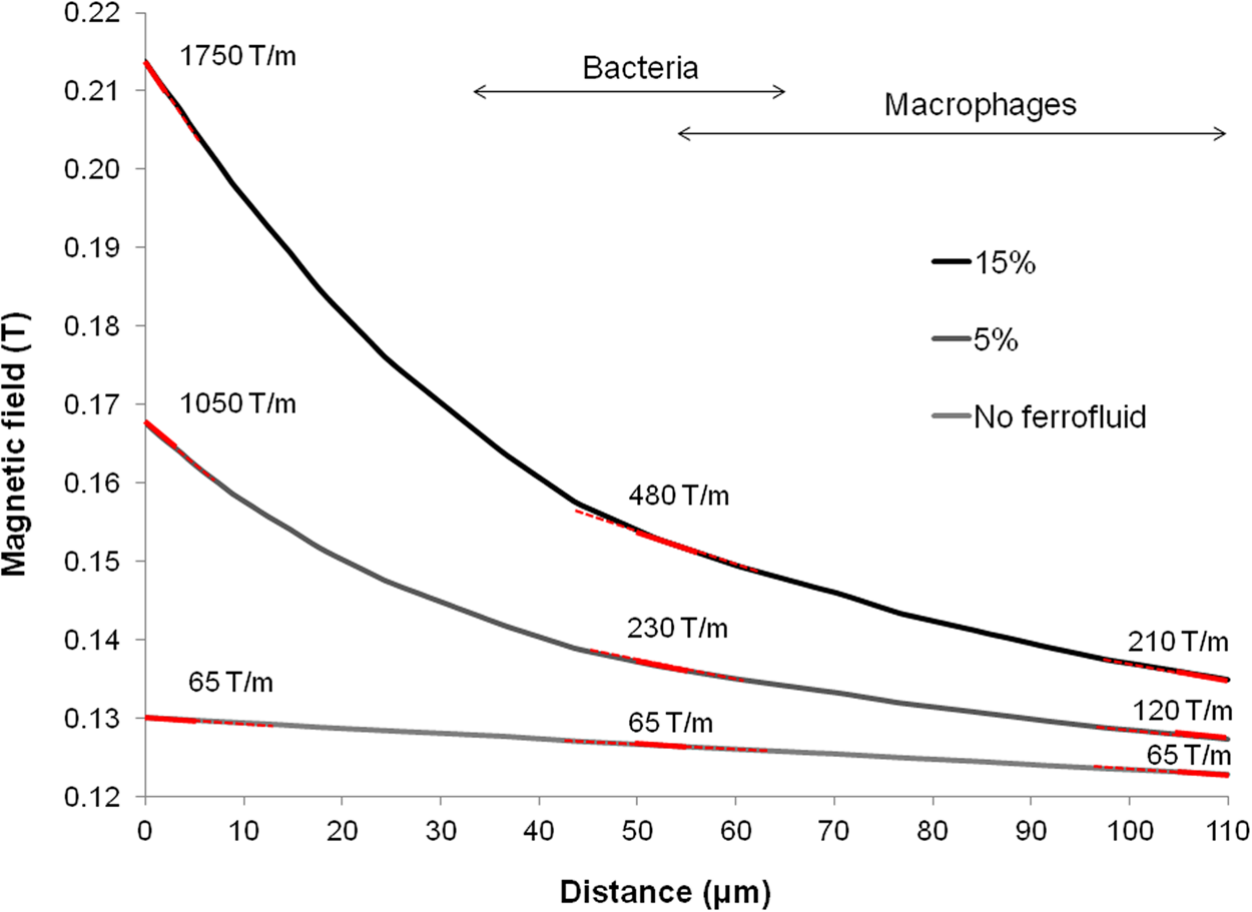
Simulations of the magnetic field in the separation channel. Estimates of the magnetic field intensity in the microfluidic channels were performed using finite elements analysis (FEMM). The graph shows the magnetic field across the sorting channel obtained for different concentrations of a ferrofluid, magnetized by a 40 MGOe NdFeB magnet. The magnetic field gradient is indicated by the red lines at three points within the sorting channel. The arrows indicate the range of distances that the bacterial or mammalian cells were covering when initially introduced into the sorting channel.

The magnetic field strength in the given microfluidic system depends mainly on the density of particles in the ferrofluid, their magnetic moment and the intensity of the applied external magnetic field. We estimated the magnetic fields with simulations using finite elements analysis (FEMM, Fig. S2), assuming the saturation magnetization for bulk magnetite (92 Am^2^/kg) in the calculation of the magnetization curves of the ferrofluid. Although a saturation magnetization of 60 Am^2^/kg was reported for 10 nm magnetite particles^30, 31^, yielding a lower magnetic moment per particle, our calculation result was in good agreement with the reported saturation magnetization of the ferrofluid used in the macrophage experiments (99 mT, Supplementary Fig. S1). The intensity of the magnetic field across the sorting channel is shown in Fig. 2; its gradient was constant at 65 T/m with the external magnet alone. Although this gradient was likely causing a small concentration profile of the ferrofluid, this effect was neglected in our simulations since its effect is small compared to the formation of chain structures which was also observed in our cell experiments. The simulations show that in the presence of a ferrofluid, the magnetic field gradient was strongly increased. The amplification effect of the ferrofluid was most evident at the wall closest to it, where a gradient of 1050 T/m and 1750 T/m was generated by a ferrofluid containing 5% and 15% magnetite particles respectively. At a distance 50 µm away from the ferrofluid, the gradients still amounted to 230 T/m and 480 T/m for the two concentrations. This distance corresponded to the initial position of the magnetic bacteria, which due to their smaller size were tested in a chip with reduced dimensions (Supplementary Fig. S3). In the case of the chip used for sorting the macrophages the magnetic field gradient was further reduced to 210 T/m (for the ferrofluid with the highest particle density), still ensuring that the cells experienced a gradient of at least three times greater than without the ferrofluid (Fig. 2).

Such an increase of the magnetic force is similar to what was obtained in previous implementations of particle-based microfluidic systems using microspheres of nickel^23^, which has a saturation magnetization (488 kA/m) similar to that of magnetite (480 kA/m). However, the non-Brownian nature of such particles (due to their larger size) would result in a smaller effect of interparticle interactions on the initial susceptibility^32^. Other metals with higher saturation magnetization such as iron (1711 kA/m) could be considered as material for a ferrofluid. On the other hand, lower magnetic core sizes than in the case of magnetite would be necessary for metallic iron to avoid phase separation so that the effect of high saturation magnetization at the material level would not translate into higher magnetic dipole moments of the magnetic cores^27^.

When the magnetic gradient in such a microfluidic system is known, one can estimate the threshold for the range of magnetic moments of samples that can be sorted for a given set of experimental parameters. By balancing the magnetic force ($m\cdot \nabla B$) and the drag force (6π*ηrv*) on a sphere in laminar flow following Stokes’ law, the crossing velocity (*v*) and thus also the time needed for a cell to travel half of the channel width can be calculated. The passage time of a cell through the channel, and thereby the interaction time with the magnetic force, is determined by the dimensions of the channel and the flow rates. By setting the crossing time equal to the passage time, the minimum magnetic moment of the sorted cells is given by

3 $$m=\frac{3\pi r\eta f}{lh\nabla B},$$

where *m* is the magnetic moment of the cell, *r* is the radius of the cell, *η* is the viscosity of the buffer, *h* and *l* are the height and length of the sorting channel, respectively, *f* is the flow rate and ($\nabla B$) is the magnetic field intensity gradient. For our system, we can estimate the minimum magnetic moments of cells that can still be sorted to be ~15 fAm^2^, assuming a cellular diameter of 10 µm (realistic for the cultured macrophages), injected at 1 µl/min (corresponding to a total flow rate 5.7 µl/min) in the presence of a 300 T/m gradient (Supplementary Fig. S4).

### Sorting of cells with intrinsic magnetic properties

**Figure 3:**
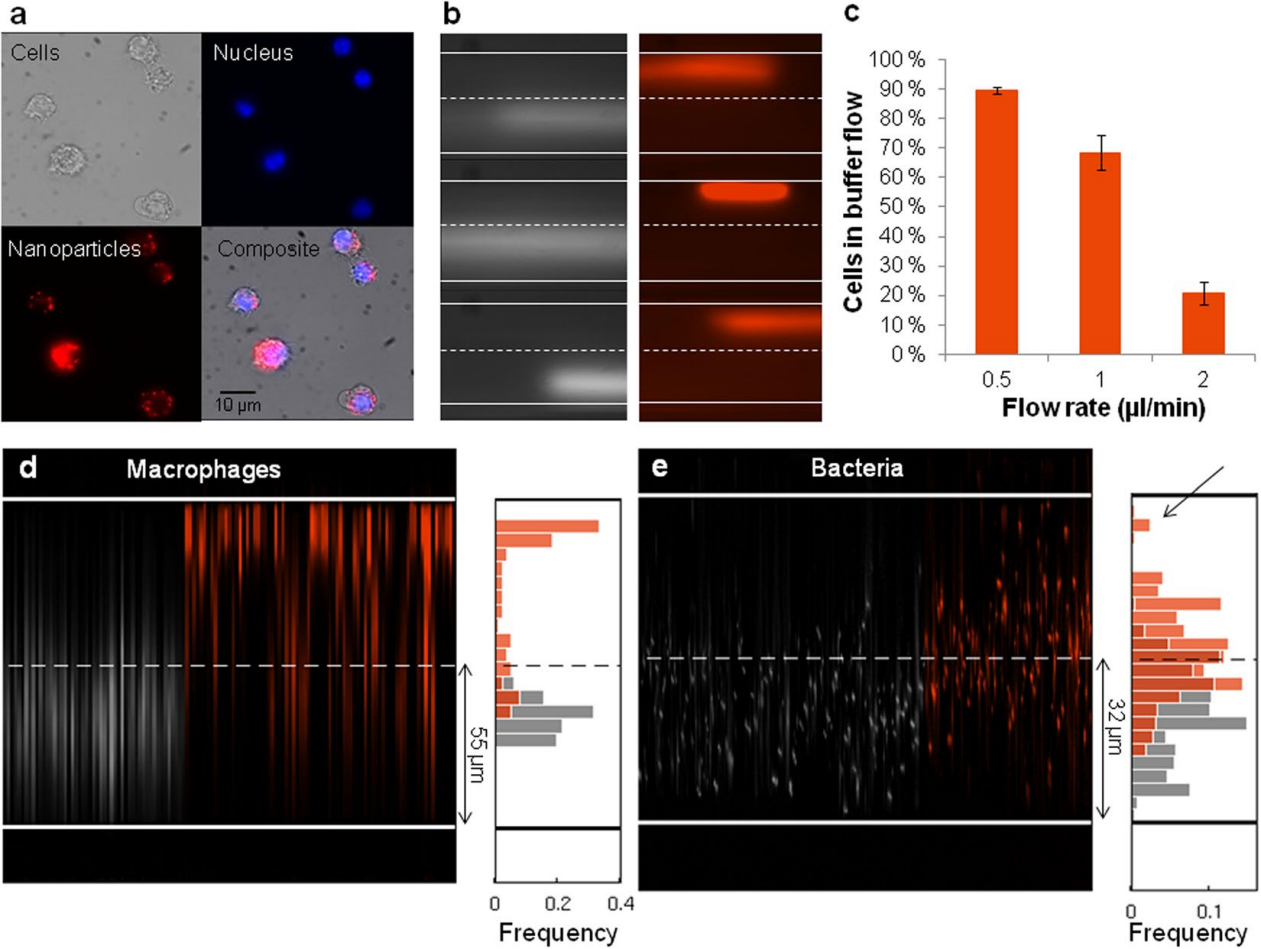
Separation of cells with intrinsic magnetic properties in the microfluidic device. (**a**) Macrophages containing phagocytosed nanoparticles (100 nm with 30 nm magnetic cores; red) were labeled with a nuclear stain (blue) for visualization in the sorting channel. (**b**) Examples of cells in the sorting channel without (gray) and with (orange) the external NdFeB magnet at a sample flow rate of 1 µl/min, observed at the center of the sorting channel. (**c**) Sorting efficiency of macrophages at different sample flow rates. The bar graph represents the average number of cells in the buffer flow with the external magnet. Error bars indicate SEM over multiple placements of the magnet (trials). (**d**) Example of data analysis for one trial of macrophages without (gray) and with (orange) the permanent magnet present. (**e**) Magnetotactic bacteria were sorted in a smaller device optimized for the smaller size of the prokaryotic cells. The histograms combines data from three trials and show a shift of the flow of bacteria in the presence (orange) of the external magnet compared to without the magnet (gray). The fraction of bacteria with highest magnetic moments was observed close to the wall (arrow).

In order to evaluate the performance of the particle-based microfluidic magnetic separation system, we first tested macrophages which were incubated with magnetite nanoparticles and labeled with a fluorescent nuclear marker for improved visualization (Fig. 3a). The macrophages contained thousands of phagocytosed magnetite nanoparticles, yielding large cellular magnetic moments on the order of 10·10^−15^ Am^2^ (corresponding to ~1500 magnetite nanoparticles per cell, or a magnetic volume concentration ~1:10^5^).

The flow of cells was observed at the center of the sorting channel (Fig. 1a,c) and the videos were analyzed by custom-written routines to evaluate the sorting efficiency, defined as the relative number of cells observed in the buffer flow (see Materials and Methods and Supplementary Fig. S5 for details). Cells were injected at 1 µl/min, while the buffer (PBS) was injected at 4.7 µl/min in order to adjust for broadening of the sample flow (due to diffusion of cells) during the experiment. By placing or removing an array of permanent magnets close to the microfluidic channels, we could reversibly magnetize the ferrofluid. In the absence of an external magnetic field only 2 ± 1% (mean ± SEM) of the cells were observed in the buffer flow, validating the presence of laminar flow conditions and allowing for an estimate of the contribution from false positives during separation. In the presence of external magnets placed in close proximity to the ferrofluid, the relative number of cells observed in the buffer flow was significantly increased to 69 ± 6% (Fig. 3b–d). In contrast, only 6 ± 1% of the cells could be sorted if no ferrofluid was present in the side channel. By further analyzing the videos, we observed that the mean position of cells in the channel was shifted by 36 pixels in the case of external magnets alone, while in the presence of ferrofluid a shift of 105 pixels was measured (Supplementary Fig. S6). This demonstrates that the magnetic force on the cells was 2.9 times greater with the ferrofluid present in the side channel compared to the magnets alone, in good agreement with the simulation results.

When the sample flow rate was increased to 2 µl/min, keeping the 1:4.7 ratio to the buffer flow rate, the relative number of cells in the buffer flow was reduced to 21 ± 4%. However, reducing the sample flow rate to 0.5 µl/min resulted in a sorting efficiency of 90 ± 1% (Fig. 3c). This demonstrated how visual control of the sorting process allowed fine-tuning of the parameters of the microfluidic system, such as the flow rate, to optimize the separation thresholds (Supplementary Fig. S4). This feature is difficult to achieve in commercial column-based sorting systems, and would also be useful for separation of heterogeneous cell populations. In this context it is also worth noting that the relatively low background magnetic field (150 mT) generated by the particles will not fully saturate other iron-rich cells which in turn could lead to an undesired enrichment^16^. The analysis could further be extended to a quantitative comparison of two or more populations of magnetic cells simultaneously by using different fluorescent markers, similar to how we characterized the cell behavior in the absence and presence of external magnets (gray and orange, respectively, in Fig. 3d,e).

Next, in order to evaluate the performance of the microfluidic magnetic separation system also for naturally occurring magnetic cells, we tested magnetotactic bacteria (*M*. *gryphiswaldense*). These bacteria are intrinsically magnetic due to their intracellular chain of magnetite nanocrystals (40 nm diameter), which however possess low magnetic moments in the range of 0.1·10^−15^ Am^2^ ^33–35^. This is just above the theoretical limit for overcoming the thermal fluctuations (*k*_*B*_ *T*) when interacting with the weak geomagnetic field (~50 µT). We tested the bacteria in a modified sorting chip that had reduced dimensions to decrease the perpendicular travel distance for the small cells (Supplementary Fig. S3). To allow for sufficient interaction time of the cells with the magnetic field gradient, cells were injected at 0.1 µl/min, while the buffer was injected at 0.5 µl/min. We observed that the mean position of the cells (visualized at the mid-point of the channel) was significantly shifted 12.1 µm towards the ferrofluid when the external magnet was present (t-test: *p* < 0.001). Specifically, as shown in the histograms in Fig. 3e, the mean position of the bacteria in the channel was 29.7 ± 0.2 µm from the wall farthest from the ferrofluid in the absence of the magnet (gray). When the external magnets were placed on the device, the mean position of the sample was 41.8 ± 0.2 µm, thus crossing the midline of the sorting channel (Fig. 3e, orange). This ensured that the bacteria were steered into the region of higher magnetic field gradient for a more efficient separation (Fig. 2). Moreover, the subpopulation of bacteria with highest magnetic volume concentration could be observed flowing along the wall close to the ferrofluid (Fig. 3e, arrow). Taken together, these results demonstrate that a large range of samples, in terms of both size and magnetic moments, could be efficiently sorted in the microfluidic system.

Visual control of the sorting process in combination with the automated video analysis was useful in the evaluation of the system and could be employed in further optimization steps. We used a fluorescent stain for improved visualization of the cells with much better contrast than available through brightfield microscopy. The approach of reducing each frame into a vertical line through a mean intensity projection along the x-axis was chosen for two reasons. First, with this approach we could easily assess the width of each object passing in the channel, thus allowing us to filter out high intensity narrow signals which are caused by cell debris or free magnetic particles in the solution. Second, when cells were flowing outside the focal plane their signal appeared weaker. These cells could be recognized by the width of the signal. Thus, our analysis allowed us to more accurately detect the number of cells by choosing the appropriate thresholds for both width and intensity of the signal (Supplementary Fig. S3). Furthermore, due to the relatively low throughput of cells, the assumption that none or only one cell is imaged in each single frame would introduce negligible errors in the estimated sorting efficiency. In the cases in which the experimental settings do not allow to have one single cell per frame (*e*.*g*. higher cell density in the medium), deviation from such assumption could be kept efficiently under control with small changes to the parameters of the analysis.

We demonstrated that separation of intrinsically magnetic cells is feasible with a simple and robust microfluidic system. Despite the limitation of low cell throughput (1000 cells/min), such a system would offer reliable and tunable separation of magnetic cell populations without the risk of introducing contaminants compromising downstream analysis. Magnetic separation of red blood cells based on their intrinsic magnetic susceptibility was first demonstrated by Melville *et al*. in 1975^36^, and sorting of red blood cells has in later years been refined through microfluidic techniques^21, 37^. Malaria infected blood cells which contain the paramagnetic crystal hemozoin exhibit a higher magnetic moment compared to uninfected blood cells, with a magnetic susceptibility of up to 320·10^−6^ per crystal^38^, corresponding to 2.5·10^−16^ Am^2^ for a crystal size of 1 µm in a magnetic field of 1 T. Thus, microfluidic magnetic isolation of infected cells for diagnostic purposes has been suggested^25, 39^. There has also been attempts to render mammalian cells paramagnetic by altering the expression of the iron storage protein ferritin^4–7^. However, in contrast to the ferrimagnetic material magnetite, the native core of (horse-spleen) ferritin is nominally an antiferromagnet with only a small fraction of uncompensated spins at the surface of the core. This results in a much weaker magnetization of 0.1 Am^2^/kg in a 1 T field^40^. In this context, the microfluidic magnetic sorting system could be valuable in a directed evolution approach^7^, where tunable enrichment of cells with a desired range of magnetic moments is important.

In summary, we here evaluated microfluidic magnetic sorting for contamination-free enrichment of cells with intrinsic magnetic properties. A ferrofluid in combination with permanent magnets could generate a strong magnetic field intensity gradient for a 10-fold increase in the separation efficiency of magnetic cells, compared to separation with a magnet alone. The system allowed for sorting of cells spanning a large range in size and magnetic moment, as demonstrated for macrophages with internalized magnetite particles as well as magnetotactic bacteria expressing magnetosome chains. Visual control and automated quantification were valuable for evaluating sorting efficiencies and fine tuning of the system parameters to achieve customized cell sorting.

## Materials and Methods

### Microfluidic chips

Microfluidic chips, compatible with microscopy, were produced by Mircronit Microtechnologies (Netherlands). The thickness of the glass above the channels was 1 mm, determining the minimum distance between the ferrofluid and external magnets. For the device used for macrophages, the dimensions of the channels were determined by the size of the mammalian cells. Thus the height of both channels was 50 µm while the width of the sorting channel was limited by production methods to minimum twice the height, and was thus 110 µm. The side channel was 150 µm wide and was filled with a ferrofluid containing 17% Vol magnetite particles with a saturation magnetization of 99 mT (EMG900, Ferrotec, Japan). In order to move the sample even closer to the ferrofluid (FER-01, Supermagnete, Germany), reduced dimensions were used for the experiment with magnetotactic bacteria. In this case, the height of the channels was 30 µm, the width of the sorting channel was 65 µm, the side channel was 100 µm wide, while all other dimensions remained the same (Supplementary Fig. S3).

### Magnetic simulations

Simulations of the magnetic field intensity were performed through a cross section of the microfluidic device using finite element analysis (D. C. Meeker, Finite Element Methods Magnetics, FEMM, version 4.2). The largest possible dimensions of the microfluidic channels (for ferrofluid and sample) were used and these were separated by 10 µm. The channels were placed 1 mm away from a sintered Nd_2_Fe_14_B permanent magnet (saturation polarization Js = 1.3 T, max. energy product 40 MGOe), which was generating a magnetizing field *H* perpendicular to the flow direction in the channels (Supplementary Fig. 2). The magnetization curve of the ferrofluid was modeled using the Langevin function with correction for particle interaction (Eqs 1 and 2, Supplementary Fig. S1) assuming a saturation magnetization of magnetite for each nanoparticle (480 kA/m). Simulations were run for particle concentrations of 5% Vol and 15% Vol as well as for the case without a ferrofluid. The magnetic field across the whole width of the sorting channel was assessed and the gradient at three different locations was estimated through linear regression across 5 µm intervals (Fig. 2).

### Sorting performance for macrophages

Cells of the murine macrophage cell line Ana-1 were grown in RPMI 1640 medium (Sigma-Aldrich, Germany) containing 10% FBS and 1% PS, incubated at 37 °C with 5% CO_2_. The macrophages were grown to confluence on 10 cm petri dishes and were incubated for 6 hours with 10 µl magnetite nanoparticles (nano-screenMAG/R Biotin 100 nm (30 nm magnetite core), Chemicell, Germany). The cells were washed twice in phosphate buffered saline (PBS) buffer to remove excess particles not phagocytosed by the cells. Macrophages were then removed from the plate using Accutase (Sigma-Aldrich, Germany), spun down at 0.4 relative centrifugal force (rcf) for 3 min, and resuspended in 3 ml PBS. Cells were labeled with a fluorescent nuclear stain (Hoechst 33342, Thermo Fisher Scientific, USA) for visualization and filtered through a cell strainer with mesh size 70 µm to avoid clogging of the microfluidic channels. The sample was diluted to ca. 1·10^6^ cells/ml in PBS containing 20% glycerol to avoid settling of cells in the syringe.

The microfluidic device was placed on an inverted microscope (Axiovert 200 M, Zeiss, Germany) using a chip holder (Fluidic Connect PRO Chipholder, Micronit Microtechnologies, Netherlands). The center of the sorting channel was imaged with fluorescence microscopy and recorded with a camera (Ximea MQ003MG-CM) at 20 fps and an exposure time of 10 ms. The cells were filled in a 1 ml syringe (Injekt, Braun, USA) and injected using a syringe pump (Elite 11, Harvard Apparatus, USA), while the buffer (PBS) was injected using a 2 ml syringe (Injekt, Braun, USA), resulting in a constant 1:4.7 ratio of the sample to buffer flow rate. The sample flow rate was adjusted to the range of 0.5–2 µl/min. Assessment of the separation efficiency in the absence of ferrofluid was performed using the same sample preparation and experimental setup with a sample flow rate of 1 µl/min. Establishment of laminar flow conditions was visually confirmed by observation of two separate flows (sample and buffer) and documented for each experiment through video recording. Three permanent magnets (NdBFe 8 × 3 × 2 mm, Magnet Shop, Germany) were placed on top of the microfluidic chip, approximately 1 mm away from the ferrofluid channel and videos were recorded. The performance of the system was evaluated for multiple placements of the external magnets to control for variability in position. For evaluation of the system and developing the quantification algorithm, we analyzed videos containing in total ~1000 cells passing the observation point, ~500 in the presence and absence of the magnet.

### Sorting of magnetotactic bacteria

Wild type magnetotactic bacteria of the strain *Magnetospirillum gryphiswaldense* (MSR-1) were grown following standard protocols^41^. The bacteria were immobilized by exposure to 75 °C for 15 minutes to avoid active swimming in the microfluidic channel, diluted in PBS buffer containing 10% glycerol and fluorescently labeled with DAPI (Sigma-Aldrich, Germany). For this experiment a chip with reduced dimensions was used (Supplementary Fig. S3). Bacterial cells were injected from a 0.5 ml syringe (Hamilton, Sigma-Aldrich, Germany) at 0.1 µl/min while the buffer PBS was injected 0.5 µl/min at the second inlet in order to adjust for broadening of the flow during the experiment, using a syringe pump (Elite 11, Harvard Apparatus, USA). The flow of cells was observed at the center of the sorting channel on an inverted microscope (Axiovert 200 M, Zeiss, Germany) and imaged with a fluorescence camera (Flea3, Point Gray, Canada) at 60 fps and an exposure time of 16 ms. In order to ensure laminar flow in the system prior to sorting, the flow was observed also in the absence of the external magnets, which were placed multiple times with one placement defining a trial. Data sets were discarded if laminar flow conditions were not observed without the magnets, ensuring observation of real sorting events.

### Data analysis

The analysis of the recorded videos for quantification of the sorting efficiency was performed using custom written code in MATLAB (Mathworks, USA) (Supplementary Fig. S5). Figure 3b shows examples of the acquired frames of macrophages in the two experimental conditions: with and without magnet. To obtain a good fluorescent signal of the cells, long exposure times were necessary during video acquisition. Thus, the cells appeared as horizontal stripes of different lengths and intensities according to the speed of the single cells. Even in laminar flow regimes of the medium, the velocities of the suspended cells vary with the velocity profile of the medium. However, in most of the frames the cells produced a continuous stripe across the whole field of view. During the experiments with macrophages, the flow rate was 1 µl/min, while the camera frame rate was 20 fps. Under these conditions the average fraction of frames containing a cell was about 0.08 (92 “empty” frames out of 100). Assuming a Poisson distribution with mean 0.08 for the number of cells in one single frame, the probability that more than one cell was imaged in a single frame is <0.003. Under such conditions, the assumption that none or only one cell is imaged in each single frame would introduce negligible errors in the estimated sorting efficiency.

In order to verify the sorting capabilities of our system and estimate the sorting efficiency, we measured the vertical shifts caused by the force acting on the cells due to the external magnetic field gradient. To this end, we estimated the vertical positions of the cells in both the experimental conditions (with and without magnet) and compared the resulting distribution histograms. First, a mean intensity projection across the x-axis was computed for each single frame to obtain an intensity profile that is a function only of the vertical position (y-axis) (Supplementary Fig. S5a). Next, a median projection along the x-axis of this image was computed and subtracted from each single column in order to correct for any background inhomogeneities (Supplementary Fig. S5b,c). After this operation, the frames that were not containing a cell were clearly detectable by eye as these columns had a flat vertical profile with low counts. In order to automatically detect the frames containing a cell, the maximum intensity and the full width half maximum (FWHM) of the single columns in the corrected image were computed (Supplementary Fig. S5d,e) and suitable filtering constrains were applied to these quantities. The position of the cells in the channel was then determined as the position along the y-axis of the peak in intensity for each of the filtered columns. The two experimental conditions could thus be conveniently compared by plotting the histograms representing the distribution of the positions of the cells. For statistical comparison of the shifts we applied the t-test. All values given are the mean with standard error (SEM).

### Data availability

The datasets generated and analyzed during the current study, as well as the MATLAB code for the quantification software, are available from the corresponding author on request.

## Electronic supplementary material

Supplementary Information 
