# Supplementary material for: Microfluidic sorting of intrinsically magnetic cells under visual control
Source: Sci Rep. 2017 Jul 31;7:6942. doi: 10.1038/s41598-017-06946-x (PMC5537260; doi:10.1038/s41598-017-06946-x)
Supplement: Supplementary file 1 — Supplementary Information [file 41598_2017_6946_MOESM1_ESM.pdf]

Microfluidic sorting of intrinsically magnetic cells under visual control

Ahne Myklatun, Michele Cappetta, Michael Winklhofer, Vasilis Ntziachristos, Gil G. Westmeyer

Supplementary material

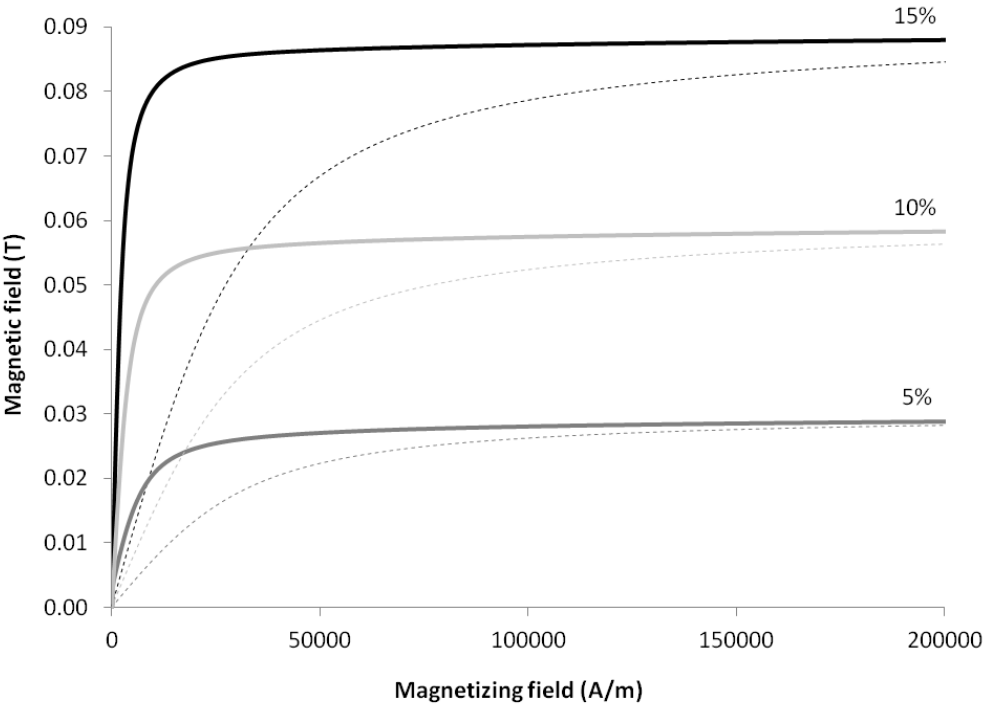

FIG S1: Magnetization curves of ferrofluids. A ferrofluid does not retain any magnetization in the absence of an external magnetic field due to the randomizing effect of the thermal energy on the single particles. However, in the presence of an external magnetic field, the magnetic moments of the particles align and magnetize the ferrofluid. The plot shows magnetization curves for ferrofluids containing different concentrations of 10 nm magnetite particles. The dotted lines represent the magnetization according to the Langevin function, while the solid lines take the correlation of particles into consideration according to equation 2; the saturation magnetization of magnetite is assumed.

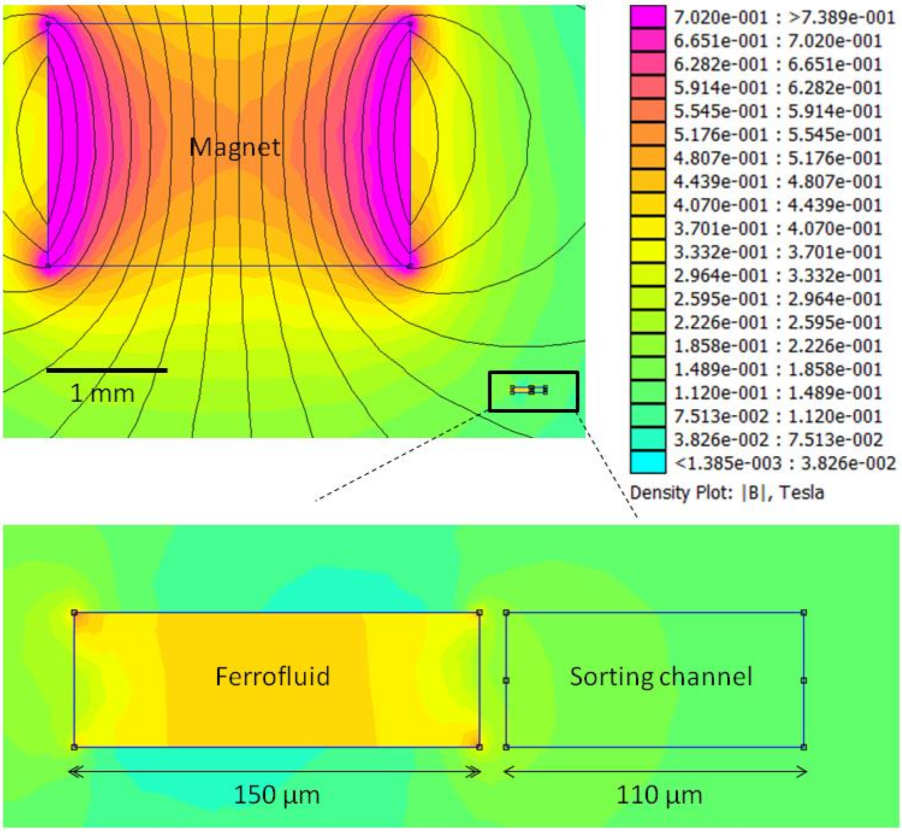

FIG S2. Magnetic field simulations. To most realistically estimate the magnetic field across the sorting channel the external magnet (NdFeB, 40 MGOe) was placed 1 mm away from sorting channel, as would be the case in our experimental setup. In this region, the magnetic field lines run orthogonal to the flow direction in the channels. FEMM (D. C. Meeker, Finite Element Method Magnetics,,version 4.2) was used for the simulation.

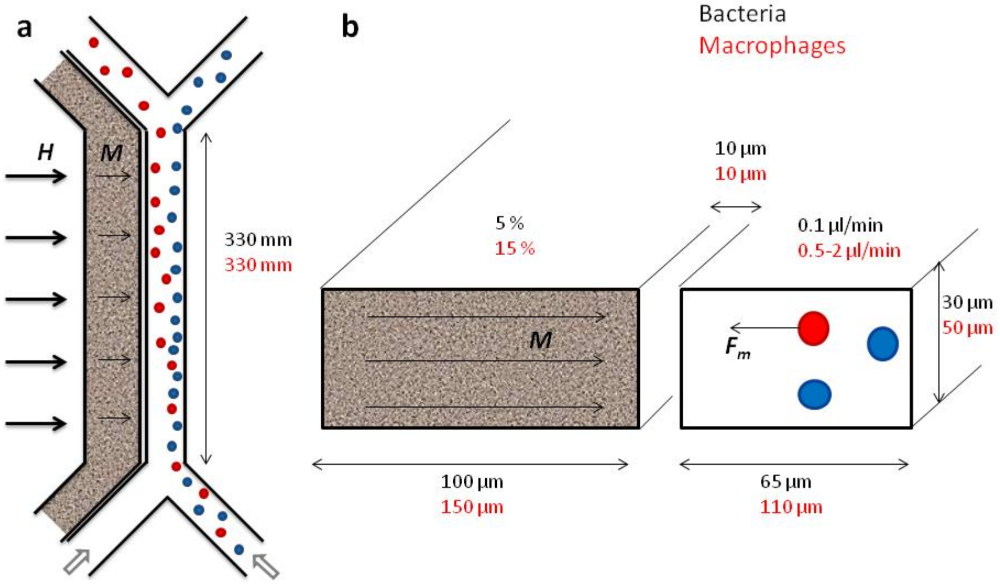

FIG. S3: Schematic of the microfluidic sorting chip. As seen from above (a) and through a cross section (b), showing the dimensions and experimental parameters used for the experiment with bacteria (black) and macrophages (red).

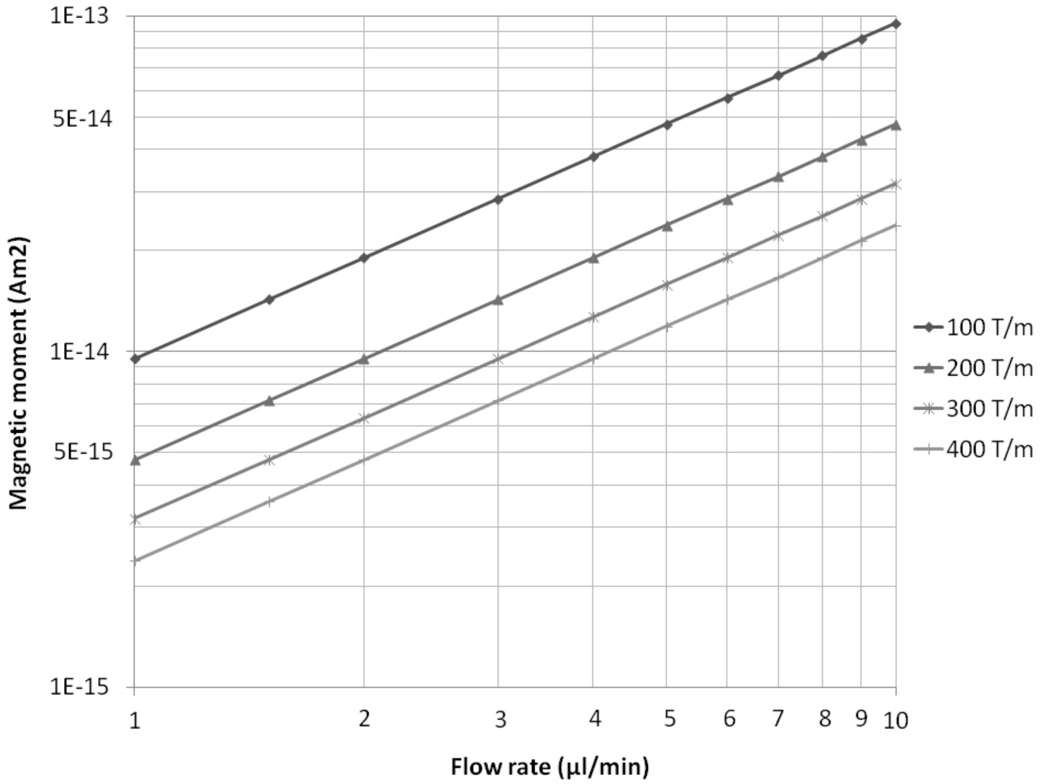

FIG. S4: Total flow rates (sum of buffer and sample) and estimates on the lower bound for magnetic moments of cells that can still be sorted with different magnetic field gradients applied and assuming a cell diameter of 10 µm.

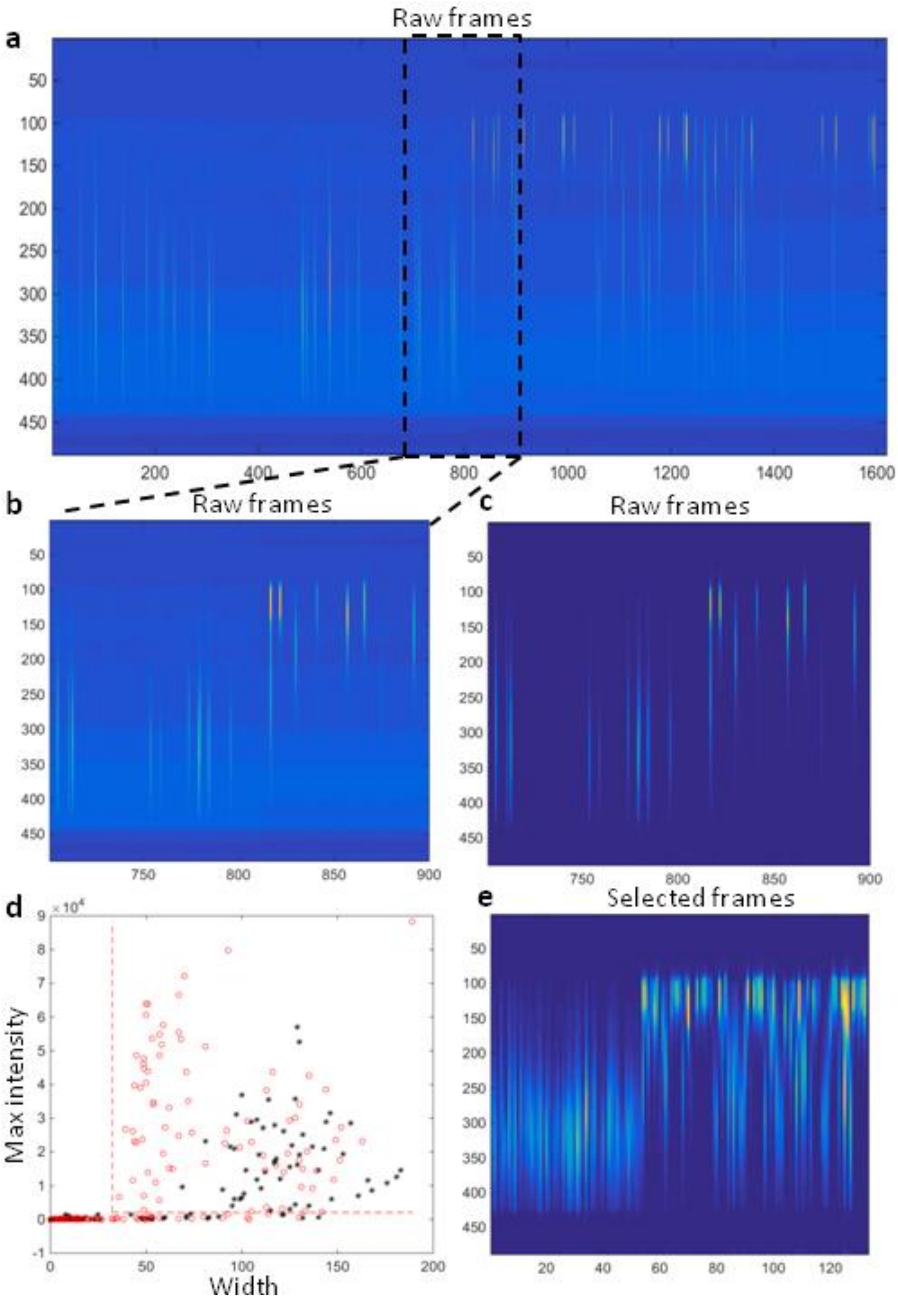

FIG. S5: Example of data analysis for quantifying the sorting efficiency. The data shown were obtained from macrophages at a sample flow rate of 1 µl/min. The different images displayed here are the raw images (a), a magnification of the transition region (b), raw frames corrected for background with the same zoom as in (c). Threshold set for automatic detection of cells (circles, black w/o magnet, red with magnet) in the images based on a minimum intensity and width of the detected fluorescent signal (red dotted lines) (d). Selected frames used for quantification (e).

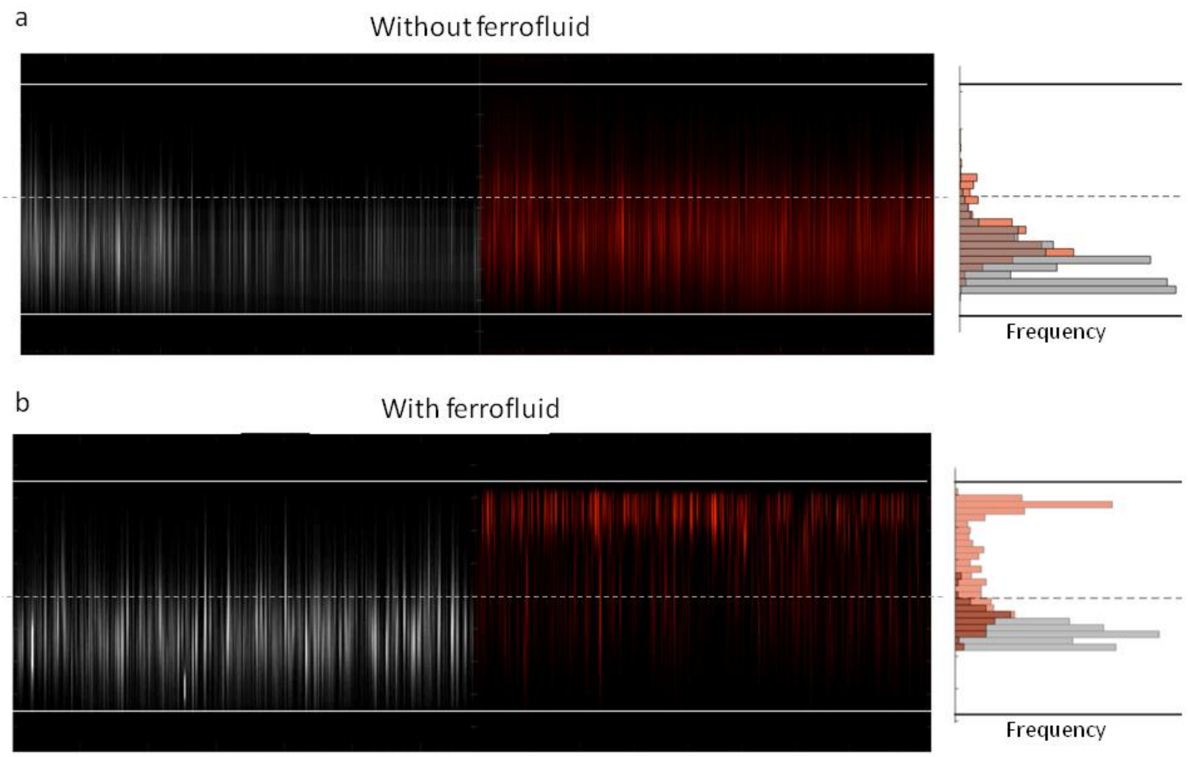

FIG. S6. Comparison of the sorting efficiency without and with ferrofluid. Macrophages with phagocytosed magnetic nanoparticles were sorted on the microfluidic chip without (gray) and with (orange) external magnet and in the absence (a) or presence (b) of the ferrofluid. The width of the channel is 110 µm, and the upper wall is facing the magnet and ferrofluid channel.
